# The *C* Allele of *ATM* rs11212617 Associates With Higher Pathological Complete Remission Rate in Breast Cancer Patients Treated With Neoadjuvant Metformin

**DOI:** 10.3389/fonc.2019.00193

**Published:** 2019-03-28

**Authors:** Elisabet Cuyàs, Maria Buxó, Maria José Ferri Iglesias, Sara Verdura, Sonia Pernas, Joan Dorca, Isabel Álvarez, Susana Martínez, Jose Manuel Pérez-Garcia, Norberto Batista-López, César A. Rodríguez-Sánchez, Kepa Amillano, Severina Domínguez, Maria Luque, Idoia Morilla, Agostina Stradella, Gemma Viñas, Javier Cortés, Jorge Joven, Joan Brunet, Eugeni López-Bonet, Margarita Garcia, Samiha Saidani, Xavier Queralt Moles, Begoña Martin-Castillo, Javier A. Menendez

**Affiliations:** ^1^Program Against Cancer Therapeutic Resistance (ProCURE), Metabolism and Cancer Group, Catalan Institute of Oncology, Girona, Spain; ^2^Girona Biomedical Research Institute (IDIBGI), Girona, Spain; ^3^Laboratori Clínic Territorial, Parque Hospitalario Martí i Julià, Salt, Spain; ^4^Breast Unit, Department of Medical Oncology, Catalan Institute of Oncology-Hospital Universitari de Bellvitge-Bellvitge Research Institute (IDIBELL), L'Hospitalet de Llobregat, Barcelona, Spain; ^5^Medical Oncology, Catalan Institute of Oncology, Girona, Spain; ^6^Medical Oncology Service, Hospital Universitario Donostia, Donostia-San Sebastián, Spain; ^7^Biodonostia Health Research Institute, Donostia-San Sebastián, Spain; ^8^Medical Oncology Department, Hospital de Mataró, Mataró, Barcelona, Spain; ^9^Hospital Quirón, IOB Institute of Oncology, Barcelona, Spain; ^10^Medical Oncology Service, Hospital Universitario de Canarias, San Cristóbal de La Laguna, Spain; ^11^Medical Oncology Service, Hospital Universitario de Salamanca, Salamanca, Spain; ^12^Instituto de Investigación Biomédica de Salamanca (IBSAL), Salamanca, Spain; ^13^Medical Oncology, Hospital Universitari Sant Joan, Reus, Spain; ^14^Medical Oncology Service, Hospital Universitario Araba, Vitoria-Gasteiz, Spain; ^15^Department of Medical Oncology, Hospital Universitario Central de Asturias, Oviedo, Spain; ^16^Department of Medical Oncology, Ramón y Cajal University Hospital, Madrid, Spain; ^17^Unitat de Recerca Biomèdica, Hospital Universitari de Sant Joan, IISPV, Rovira i Virgili University, Reus, Spain; ^18^Hereditary Cancer Programme, Catalan Institute of Oncology (ICO), Bellvitge Institute for Biomedical Research (IDIBELL), L'Hospitalet del Llobregat, Barcelona, Spain; ^19^Hereditary Cancer Programme, Catalan Institute of Oncology (ICO), Girona Biomedical Research Institute (IDIBGI), Girona, Spain; ^20^Department of Anatomical Pathology, Dr. Josep Trueta Hospital of Girona, Girona, Spain; ^21^Clinical Research Unit, Catalan Institute of Oncology, L'Hospitalet de Llobregat, Barcelona, Spain; ^22^Unit of Clinical Research, Catalan Institute of Oncology, Girona, Spain

**Keywords:** metformin, breast cancer, neoadjuvancy, HER2, ATM, *rs11212617*

## Abstract

**Background:** The minor allele (*C*) of the single-nucleotide polymorphism (SNP) *rs11212617*, located near the *ataxia telangiectasia mutated* (*ATM*) gene, has been associated with an increased likelihood of treatment success with metformin in type 2 diabetes. We herein investigated whether the same SNP would predict clinical response to neoadjuvant metformin in women with early breast cancer (BC).

**Methods:** DNA was collected from 79 patients included in the intention-to-treat population of the METTEN study, a phase 2 clinical trial of HER2-positive BC patients randomized to receive either metformin combined with anthracycline/taxane-based chemotherapy and trastuzumab or equivalent regimen without metformin, before surgery. SNP *rs11212617* genotyping was assessed using allelic discrimination by quantitative polymerase chain reaction.

**Results:** Logistic regression analyses revealed a significant relationship between the *rs11212617* genotype and the ability of treatment arms to achieve a pathological complete response (pCR) in patients (odds ratio [OR]_genotype×arm_ = 10.33, 95% confidence interval [CI]: 1.29–82.89, *p* = 0.028). In the metformin-containing arm, patients bearing the *rs11212617 C* allele had a significantly higher probability of pCR (OR_*A*/*C,C*/*C*_ = 7.94, 95%CI: 1.60–39.42, *p* = 0.011). Conversely, no association was found between *rs11212617* and clinical response in the reference arm (OR_*A*/*C,C*/*C*_ = 0.77, 95%CI: 0.20–2.92, *p* = 0.700). After controlling for tumor size and hormone receptor status, the *rs11212617 C* allele remained a significant predictor of pCR solely in the metformin-containing arm.

**Conclusions:** If reproducible, the *rs11212617 C* allele might warrant consideration as a predictive clinical biomarker to inform the personalized use of metformin in BC patients.

**Trial Registration:** EU Clinical Trials Register, EudraCT number 2011-000490-30. Registered 28 February 2011, https://www.clinicaltrialsregister.eu/ctr-search/trial/2011-000490-30/ES.

## Introduction

The minor allele *C* of the noncoding single nucleotide polymorphism (SNP) *rs11212617*, which is located near the *ataxia telangiectasia mutated* (*ATM*) gene, was found to be associated with the metabolic response to the biguanide metformin in the first genome-wide association study (GWAS) carried out in 3,912 Europeans with type 2 diabetes (T2D) (1). Although lack of replication occurred in some studies aiming to verify the association between *rs11212617* and the effect of metformin in multiple ethnic groups (2), a meta-analysis in smaller cohorts suggested that the *rs11212617 C* allele might be considered as the first robustly replicated common susceptibility locus associated with metformin treatment success in patients with T2D (3). Moreover, *rs11212617* remained a top signal with no other genome-significant hits in a more recent GWAS of 13,123 participants of different ancestries, but failed to associate with glycemic response to metformin in a systematic three-stage replication study (4). However, *rs11212617* has recently been shown to significantly affect not only the response to metformin in terms of insulin Z score, but also metformin plasma concentration (5). Mechanistic studies have shown that *rs11212617* increases enhancer activity and could lead to elevated expression of several target genes including *ATM* itself (6). Yet, almost nothing is known about the impact of the *rs11212617 C* allele on the clinical efficacy of metformin in several ongoing clinical trials aiming to evaluate its potential benefits in a cancer setting (7).

A potential anti-cancer effect of metformin has gained considerable epidemiological and pre-clinical support over the last decade (7–10). First, a large number of population-based observational and cohort studies have suggested a cancer-preventive advantage associated with metformin usage among T2D patients (11). Second, diabetic patients with breast cancer receiving metformin during neoadjuvant chemotherapy were reported to benefit from a 3-fold greater pathological complete response (pCR) when compared with those who did not receive metformin (12). Third, an ever-growing number of pre-clinical studies have proposed numerous cell-autonomous (e.g., AMPK/mTOR-related) and non-cell-autonomous (e.g., insulin/IGF-1-related) molecular mechanisms that have enthusiastically endorsed the clinical development of metformin as a novel anti-cancer drug (13–15). However, one should acknowledge that a metformin-driven cancer-preventive advantage does not necessarily imply an effective therapeutic efficacy in non-diabetic patients with established cancers, and it remains unclear whether the adjuvant use of metformin in combination with standard cancer therapy could translate into better clinical outcomes (16–19). Indeed, recent randomized studies reporting the use of metformin in cancer treatment have yielded mixed results in patients with advanced disease (20, 21). Although the results of much larger randomized studies, such as NCIC CTG MA.32, the most advanced adjuvant trial investigating the effects of metformin vs. placebo on invasive disease-free survival and other outcomes on early breast cancer in 3,649 women (22), will be of great interest to confirm or reject the causal nature of the suggested correlation between metformin use and survival benefit in cancer patients, it is also true that companion biomarker studies are urgently needed to refine tumor and patient selection when using metformin as an adjuvant to established cancer therapeutics.

We herein investigated whether the presence of the *rs11212617 C* allele could predict the pathological complete response (pCR) in the METTEN study (23, 24), a randomized, open-label, multicenter, phase 2 trial of neoadjuvant metformin in combination with trastuzumab and chemotherapy in women with early HER2-positive breast cancer.

## Materials and Methods

### Subjects

The METTEN study was registered with the EU Clinical Trials Register and is available online (https://www.clinicaltrialsregister.eu/ctr-search/trial/2011-000490-30/ES). Patients were randomly assigned to receive daily metformin (850 mg twice-daily) for 24 weeks concurrently with 12 cycles of weekly paclitaxel (80 mg/m^2^) plus trastuzumab (4 mg/kg loading dose followed by 2 mg/kg) followed by four cycles of 3 weekly fluorouracil (600 mg/m^2^), epirubicin (75 mg/m^2^), cyclophosphamide (600 mg/m^2^) with concomitant trastuzumab (6 mg/kg) (arm A), or equivalent sequential chemotherapy plus trastuzumab without metformin (arm B), followed by surgery. Patients had surgery within 4–5 weeks of the last cycle of neoadjuvant treatment (24). Post-surgery, patients received thrice-weekly trastuzumab to complete 1 year of neoadjuvant-adjuvant therapy. Genotyping of SNP *rs11212617* was carried out in the intention-to-treat (ITT) population (*n* = 79), which included all randomly assigned patients who received at least one dose of study medication.

### Assessment of Pathological Complete Response (pCR)

pCR was defined as absence of invasive tumor cells on hematoxylin and eosin evaluation of the complete resected breast specimen (and all sample regional lymph nodes if lymphadenectomy was performed) following the completion of neoadjuvant systemic therapy. Residual ductal carcinoma *in situ* (DCIS) only was included in the definition of pCR (ypT0/is, ypN0) (24).

### Analytical Methods

Blood was drawn after an overnight fast. Serum glucose was measured in duplicate using the glucose oxidase method and serum insulin was measured in duplicate using the Human Insulin ELISA (Cat. # EZHI-14K, Merck Millipore, Billerica, MA). The lowest level of insulin that can be detected by this assay is 2 μU/mL when using a 20 μL sample size. Intra- and inter-assay coefficients of variation were below 6 and 11%, respectively. Fasting insulin resistance was calculated using the homeostasis model assessment (HOMA) using the following formula: HOMA-IR = fasting glucose (mmol/L) × fasting insulin (mU/L)/22.5.

### Genotyping of SNP rs11212617

The *ATM rs11212617* SNP variants were determined using the 5′ exonuclease TaqMan-based allelic discrimination method (Applied Biosystems, assay ID C_134213_10).

### Statistical Analysis

Descriptive data were summarized using percentages, medians or means with their respective 25 and 75 percentiles or standard deviations as appropriate. Clinical baseline characteristics between groups (non-pCR and pCR) were assessed using Chi-square or Fisher's exact test for categorical variables, student *t-*test for continuous variables with normal distribution, or Mann-Whitney *U* test for non-normal distributions. The assumption of normality was evaluated with the Shapiro-Wilk test. Changes in glucose, insulin, and HOMA-IR between pre and post treatment were compared using the Wilcoxon test. The R package Hardy-Weinberg (http://www.jstatsoft.org/v64/i03/) was employed to check whether the Hardy-Weinberg equilibrium holds among study population. Binary logistic regression was used to assess the prognostic effect of baseline *rs11212617* genotype on pCR. Unadjusted and adjusted odds ratios (ORs) with their relative 95% confidence intervals (CIs) were reported as a measure of association. All tests were 2- sided and *P* ≤ 0.05 was set as statistically significant. Statistical analyses were carried out using SPSS (IBM Corp. released 2017. IBM SPSS Statistics for Windows, Version 25.0; Armonk, NY) and STATA (StataCorp. 2013. Stata Statistical Software: Release 13; StataCorp LP, College Station, TX).

## Results

### Study Participants

**Figure 1:**
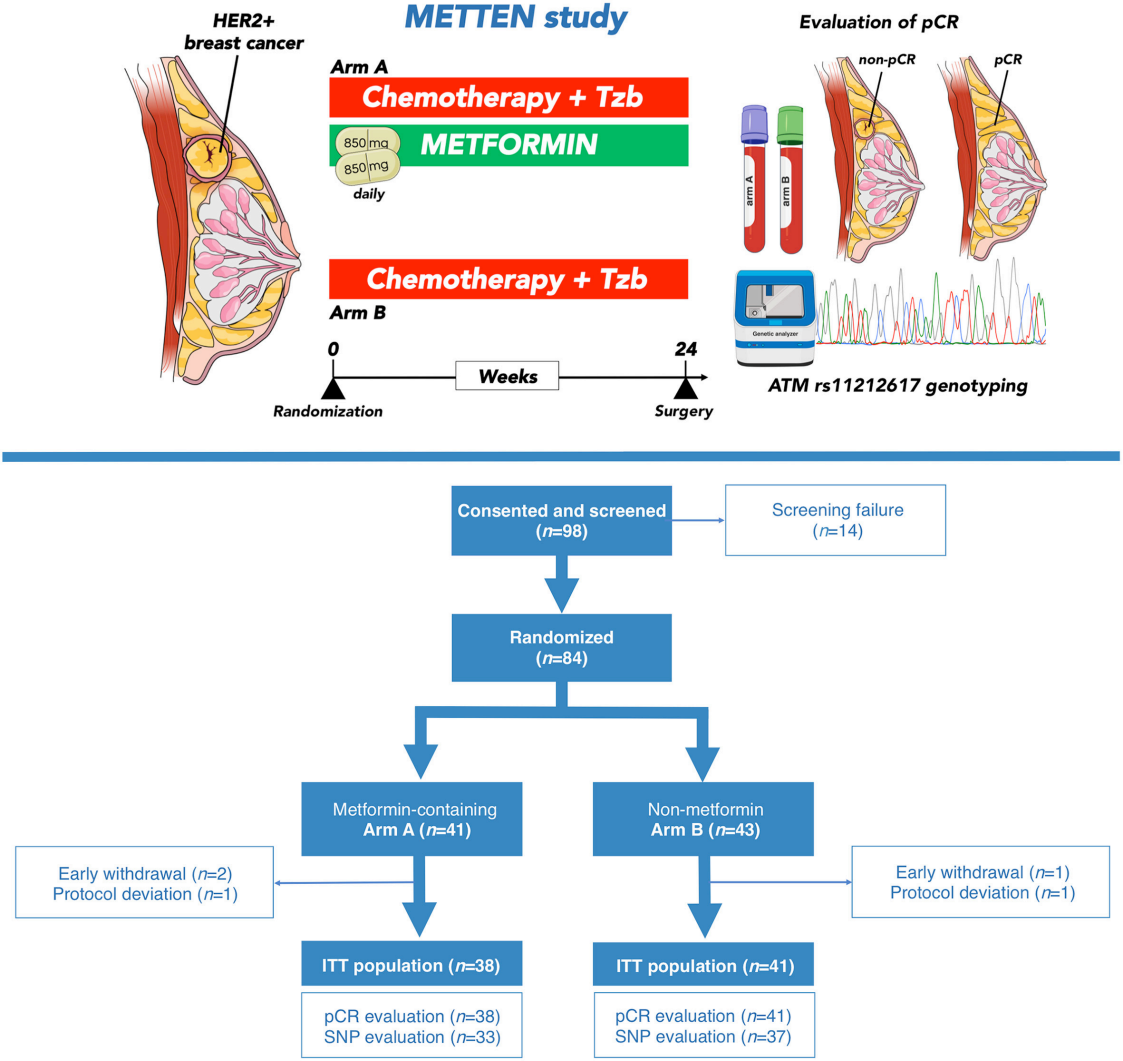
The METTEN study. **(Top)** The open-label, multicenter, phase II randomized METTEN study was designed to evaluate the clinical activity, tolerability, and safety of adding metformin to neoadjuvant chemotherapy plus trastuzumab in operable, locally advanced, or inflammatory HER2-positive BC (23, 24). Women with primary, non-metastatic HER2-positive BC were randomly assigned (1:1) to receive daily metformin (850 mg twice-daily) for 24 weeks concurrently with 12 cycles of weekly paclitaxel plus trastuzumab followed by four cycles of 3 weekly fluorouracil, epirubicin, cyclophosphamide plus trastuzumab (arm A) or equivalent sequential chemotherapy plus trastuzumab without metformin (arm B), followed by surgery. The primary end point was pCR, defined as absence of invasive tumor cells on hematoxylin and eosin evaluation of the complete resected breast specimen (and all sample regional lymph nodes if lymphadenectomy was performed) following the completion of neoadjuvant systemic therapy. Residual ductal carcinoma *in situ* (DCIS) only was included in the definition of pCR (ypT0/is, ypN0). Between June 1, 2012 and March 17, 2016, 98 patients at 10 centers in Spain were recruited into the METTEN study. DNA sample collection was not included in the original study design and was added as addendum #3 in April 2012 to re-consent patients for an additional blood draw for germ line DNA extraction. DNA samples from 70 patients (89% of the full ITT cohort) were subsequently collected and genotyped for SNP *rs11212617*. **(Bottom)** Modified CONSORT diagram showing the 70 cases of HER2-positive BC patients used for the analysis of clinical response analysis to neoadjuvant metformin by the minor allele C of the SNP *rs11212617*.

This study was designed to evaluate the clinical relevance of the SNP *rs11212617 C* allele with respect to its potential to predict a pCR in breast cancer patients with HER2 overexpression treated with metformin-containing neoadjuvant systemic therapy (Figure 1). We conducted the study with patients belonging to the ITT population of the METTEN trial, which included all randomly assigned patients who received at least one dose of study medication (*n* = 79) (24). A flowchart describing the formation of each cohort in the study is shown in Figure 1. The baseline characteristics of those ITT patients who achieved pCR after neoadjuvant therapy and those who did not are shown in Table 1. The comparison of clinical-pathological variables at diagnosis between patients of each non-pCR/pCR cohort revealed no significant differences, except for hormone receptor status. The non-pCR group tended to have more estrogen receptor-negative and/or progesterone-positive tumors (*p* = 0.056).

**Table 1:** Clinical characteristics of patients at baseline according to pathological complete response (pCR) status.

	**Non-pCR (*n* = 31)**	**pCR (*n* =48)**	***p*-value**
**Arm**			0.335
A	17 (54.8%)	21 (43.8%)	
B	14 (45.2%)	27 (56.3%)	
**SNP rs11212617**^a^			0.214^*1^
*A/A*	18 (64.3%)	19 (45.2%)	
*A/C*	6 (21.4%)	17 (40.5%)	
*C/C*	4 (14.3%)	6 (14.3%)	
			0.118
*A/A*	18 (64.3%)	19 (45.2%)	
*A/C, C/C*	10 (35.7%)	23 (54.8%)	
**Age**			0.465
<50	20 (64.5%)	27 (56.3%)	
≥50	11 (35.5%)	21 (43.8%)	
Mean ± SD (min;max)	47.1 ± 11.9 (30;75)	48.0 ± 10.6 (23;71)	0.741
**Premenopausal status**			0.583
Post	11 (35.5%)	20 (41.7%)	
Pre+Peri	20 (64.5%)	28 (58.3%)	
**Body weight (kg)**			
Mean ± SD (min;max)	64.3 ± 6.9 (48;78)	65.3 ± 10.2 (45.3;89.0)	0.592
**Body-mass index**			0.179
<25	19 (61.3%)	22 (45.8%)	
≥25 (overweight)	12 (38.7%)	26 (54.2%)	
**Clinical tumor status**			0.077^*1^
cT2	18 (58.1%)	33 (68.8%)	
cT3	12 (38.7%)	10 (20.8%)	
cT4a	1 (3.2%)	0 (0.0%)	
cT4b	0 (0.0%)	4 (8.3%)	
cT4d	0 (0.0%)	1 (2.1%)	
**Clinical nodal stage**			0.581^*1^
cN0	6 (19.4%)	16 (33.3%)	
cN1	20 (64.5%)	24 (50.0%)	
cN2	1 (3.2%)	2 (4.2%)	
cN2a	1 (3.2%)	0 (0.0%)	
cN2b	0 (0.0%)	1 (2.1%)	
cN3	3 (9.7%)	4 (8.3%)	
cN3c	0 (0.0%)	1 (2.1%)	
**Hormone receptor status**			0.056
ER and/or PgR positive	21 (67.7%)	22 (45.8%)	
ER and PgR negative	10 (32.3%)	26 (54.2%)	
**Tumor grade**^b^			1.000^*1^
G1	1 (4.0%)	1 (2.8%)	
G2	12 (48.0%)	18 (50.0%)	
G3	12 (48.0%)	17 (47.2%)	
**Baseline glucose (mmol/L)**			
Mean ± SD (min;max)	5.2 ± 0.4 (4.4;6.0)	5.2 ± 0.5 (3.9;6.5)	0.511
**Baseline insulin (mU/mL)**			
Mean ± SD (min;max)	8.7 ± 12.2 (2.1;62.9)	8.2 ± 5.2 (3.0;21.6)	0.834
**Baseline HOMA**			
Mean ± SD (min;max)	1.9 ± 2.6 (0.5;13.1)	1.9 ± 1.2 (0.6;5.1)	0.964

*1 *Fisher exact test*.

a *Data available for 70 of 79 patients*.

b *Data available for 61 of 79 patients*.

### Allele Frequencies of *rs11212617*

The *rs11212617* polymorphism was evaluable in most of the patient samples, and 70 of 79 patients (89%) were genotyped (Figure 1, Table 2). The *A* and *C* allelic frequencies of *rs11212617* in our patients were 69 and 31%, respectively. The frequencies of three genotypes in all the patients were 14.3% (*C/C*), 32.9% (*A/C*), and 52.9% (*A/A*). These genotype frequencies were very similar to those predicted by the Ensembl genome database for a Tuscany, in Italy (TSI) population, and slightly different to those observed in Europeans and the Iberian population in Spain (Table 2). Despite the small population size, there was no significant deviation in *rs11212617* genotype frequencies in our population from the Hardy-Weinberg expectation [HWE; Sum Equally Likely or More Extreme [SELOME] *p* = 0.0879]. No significant differences were observed in the genotype frequencies of SNP *rs11212617* between the non-pCR and pCR cohorts in the ITT population (Table 1).

**Table 2:** Expected and observed SNP rs11212617 prevalence (%).

	**Expected^a^**			**Observed**	
	**IBS^b^**	**EUR^c^**	**TSI^d^**	**METTEN trial (*****n*** **=** **70)**	
**ATM *rs11212617***	***%***	***%***	***%***	***n***	***%***
*A/A*	41.1	38.2	51.4	37	52.9
*A/C*	49.5	47.1	35.5	23	32.9
*C/C*	9.3	14.7	13.1	10	14.3

a *http://www.ensembl.org/Homo_sapiens/Variation/Population?db=core;r = 11:108411934-108412934;v = rs11212617;vdb = variation;vf = 6530681#373524_tablePanel*.

b *IBS, Iberian Population in Spain*.

c *EUR, European*.

d *TSI, Tuscany in Italy*.

### Association Between *rs11212617* and Clinical Response

**Figure 2:**
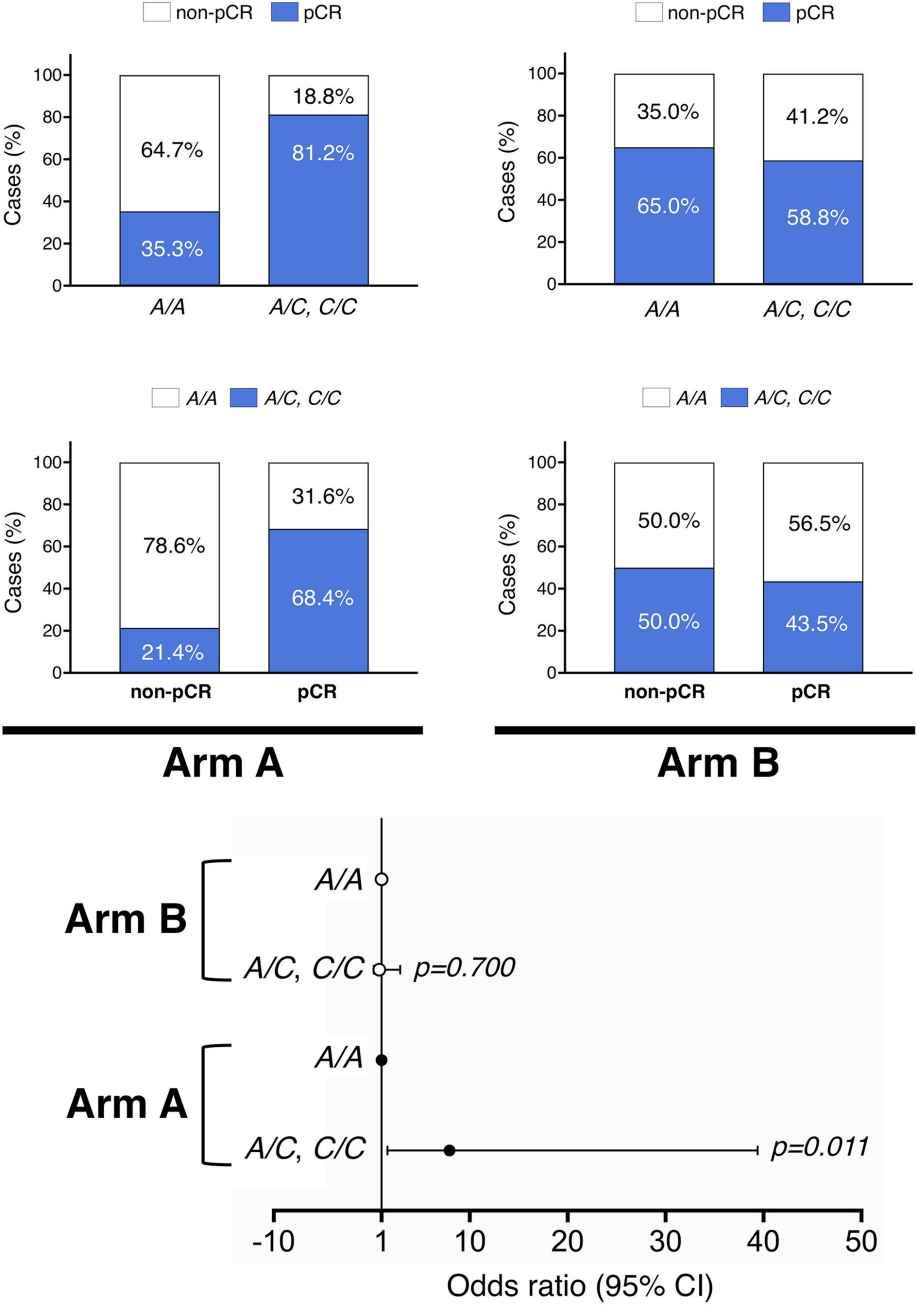
Association of ATM *rs11212617* genotype and pCR by treatment arm. **(Top)** Rates of pCR stratified by the (C) *rs11212617* genotype in patients randomized to receive either metformin combined with anthracycline/taxane-based chemotherapy and trastuzumab (arm A) or equivalent regimen without metformin (arm B). **(Bottom)** Relationship between the (C) *rs11212617* genotype and the ability of treatment arms to achieve pCR.

Frequency distributions of SNP *rs11212617* were similar between treatment arms (Table S1). Of the patients in the metformin-containing arm A, 81.2% of homo or heterozygous patients for the *rs11212617 C* allele achieved a pCR, whereas 64.7% of non-carrier patients did not achieve a pCR (Figure 2, top panels). Of the patients in the reference arm B, 58.8% of homo or heterozygous patients for the *rs11212617 C* allele and 65% of non-carrier patients achieved a pCR, respectively (Figure 2, top panels). We employed logistic binary regression analyses to investigate the association between arm, *ATM rs11212617* genotype, and pCR. In bivariate analysis, we failed to show predictive capacity of either the arm treatment or *rs11212617* genotype with the probability of achieving pCR (Table S2). However, we observed a significant relationship between *rs11212617* genotype and the ability of treatment arms to achieve pCR (OR_genotype×arm_ = 10.33, 95%CI: 1.29–82.89, *p* = 0.028; Table 3). This finding suggested that the direction and/or intensity of the relationship between *rs11212617* genotype and pCR significantly varied in each treatment arm. Accordingly, the patients bearing the *rs11212617 C* allele in the metformin-containing arm had a significantly higher probability of pCR (OR_*A*/*C, C*/*C*_ = 7.94, 95%CI: 1.60–39.42, *p* = 0.011; Figure 2, bottom panel). Conversely, no association was found between the presence of the *rs11212617 C* allele and clinical response in the (non-metformin) reference arm (OR_*A*/*C, C*/*C*_ = 0.77, 95%CI: 0.20–2.92, *p* = 0.700; Figure 2, bottom panel). After additional adjusting for potential confounding tumor characteristics such as tumor size and hormone receptor (HR) status, a relationship between the *rs11212617* genotype and the ability of treatment arms to achieve a pCR in patients remained significant (adjusted OR_genotype×arm_ = 20.53, 95%CI: 1.97–213.79, *p* = 0.011; Table S3). In the metformin-containing arm, the positive association between the presence of the *rs11212617 C* allele and pCR remained significant after accounting for tumor size and HR status (adjusted OR_*A*/*C, C*/*C*_ = 28.88, 95%CI: 2.20–378.73, *p* = 0.010; Table S4). The lack of association between the *rs11212617 C* allele and pCR in the (non-metformin) reference arm was not altered after adjusting for these factors (Table S5).

**Table 3:** Association of the interaction between ATM *rs11212617* genotype and pCR by treatment arm.

		**Odds ratio (95% CI)**	***p*-value**
ATM *rs11212617*	*A/A*	1	
	*A/C, C/C*	0.77 (0.20–2.92)	0.700
Arm	B	1	
	A	0.29 (0.08–1.14)	0.076
Genotype × Arm		10.33 (1.29–82.99)	0.028

### Association Between ATM rs11212617 and Metabolic Response

A Wilcoxon test was conducted to evaluate whether there was a significant relationship between the *rs11212617 C* allele and the metabolic response to each arm. In the reference arm, no significant relationship between *rs11212617 C* allele and reductions in glucose, insulin, or HOMA-IR index was evident (Table 4). In the metformin arm, however, there was a near-significant trend between the *rs11212617 C* allele and the metabolic response to metformin in terms of insulin reduction (*p* = 0.069; Table 4).

**Table 4:** Association of ATM *rs11212617* genotype with changes in glucose, insulin, and HOMA-IR pre- and post-treatment.

		**Pre-treatment**	**Post-treatment**	***p*-value^a^**			**Pre-treatment**	**Post-treatment**	***p*-value^a^**
**Arm A**	*n*	MD^b^ (p25,p75)	MD (p25,p75)		**Arm B**	*n*	MD (p25,p75)	MD (p25,p75)	
**Glucose**					**Glucose**				
*A/A*	17	5.27 (4.99,5.54)	5.38 (5.13,5.66)	0.410	*A/A*	20	5.14 (4.82,5.30)	5.13 (4.60,5.77)	0.588
*A/C,C/C*	16	5.26 (4.84,5.43)	5.03 (4.79,5.36)	0.109	*A/C,C/C*	17	5.30 (4.84,5.50)	5.30 (4.92,5.64)	0.344
**Insulin**					**Insulin**				
*A/A*	14	4.78 (3.76,6.39)	7.22 (2.73,8.95)	0.245	*A/A*	9	5.55 (3.59,11.68)	7.85 (4.93,17.60)	0.441
*A/C,C/C*	8	6.38 (3.56,9.54)	4.39 (2.26,6.75)	0.069	*A/C,C/C*	10	6.46 (3.82,10.43)	4.27 (3.74,7.21)	0.169
**HOMA-IR^c^**					**HOMA-IR**				
*A/A*	14	1.13 (0.97,1.55)	1.51 (0.64,2.10)	0.397	*A/A*	9	1.10 (0.80,2.79)	1.96 (0.94,5.36)	0.260
*A/C,C/C*	8	1.54 (0.82,2.25)	0.95 (0.46,1.65)	0.093	*A/C,C/C*	10	1.45 (0.86,2.61)	1.09 (0.80,1.80)	0.241

a *Wilcoxon test*.

b *MD, Median*.

c *Homeostasis Model Assessment of Insulin Resistance*.

## Discussion

A significant number of neoadjuvant, adjuvant, and advanced disease trials are currently ongoing or have been proposed to elucidate whether metformin, when used at doses established for diabetes control, has the potential to be used in preventive and treatment settings as an adjuvant to established cancer therapeutics. In this scenario, companion biomarker studies are urgently needed to define metformin efficacy and refine the tumor types and/or patient populations that are most likely to benefit from metformin-containing interventions.

To our knowledge, this is the first prospective study evaluating the relationship between the *ATM* SNP rs11212617 *C* allele, which has been associated with an increased likelihood of metformin treatment success in T2D (1, 3, 5), and the clinical benefit of adding metformin to well-established neoadjuvant treatment regimens in breast cancer patients. Logistic regression analyses revealed a significant relationship between the *rs11212617* genotype and the ability of treatment arms to achieve a pCR. In the metformin-containing arm, patients bearing the *rs11212617 C* allele had a significantly higher probability of pCR. Conversely, no association was found between *rs11212617* and clinical response in the reference arm. Because greater benefits from HER2-targeted neoadjuvant treatment in breast cancer are achieved in patients with small HR-negative tumors compared with patients with large HR-positive tumors (25), it is noteworthy that the capacity of the ATM *rs11212617 C* allele to predict a higher chance of achieving a pCR in patients treated with neoadjuvant metformin was not altered after accounting for factors like tumor size and HR status.

A previous report by Reni et al. (21) failed to observe any association between the *C* allele of *rs11212617* and the clinical response to metformin in pancreatic cancer, but a significant relationship between the highest reduction of fasting plasma glucose and the *CC* genotype was observed. Our study suggests that the presence of the minor *C* allele of *rs11212617* might associate with a significant improvement in insulin sensitivity in HER2-positive breast cancer patients subjected to neoadjuvant metformin in combination with trastuzumab and chemotherapy. This was evidenced by a near significant reduction of circulating insulin levels and HOMA-IR index—which fairly correlates with the insulin sensitivity index calculated using the minimal model approach (26), solely in those patients bearing SNP *rs11212617 C* allele in the metformin-containing arm despite maintenance of blood glucose levels.

Limitations of this study are inherent in the design; in particular, the open-label nature of the study, and a relatively modest sample size. Further, because a concurrent analysis of well-characterized breast cancer biomarkers relevant for the putative mechanism of metformin was not achievable, it might be argued that the outcome predicted by the “favorable” *C* allele could be partially biased. Cancer cells expressing constitutively active phosphatidylinositol-3 kinase (PI3K) are proliferative regardless of the absence of insulin, and they can form dietary restriction (DR)-resistant tumors *in vivo* (27). Accordingly, because the binding of insulin to its receptors activates the PI3K/AKT/mammalian target of rapamycin (mTOR) signaling cascade, activating mutations in the *PIK3CA* oncogene might be expected to determine tumor response to DR-like pharmacological strategies targeting the insulin and mTOR pathways (27, 28). In our hands, however, breast cancer xenografts harboring the insulin-unresponsive, DR-resistant, *PIK3CA*-activating mutation H1047R remained largely sensitive to the anti-tumoral effects of metformin (29). Given that new groundbreaking research has shown how dietary approaches such as carb-restricted ketogenic diets can prevent the systemic glucose-insulin feedback that impairs the efficacy of PI3K inhibitors (30), our current findings, together with the ability of metformin to significantly augment the circulating the levels of the ketone body beta-hydroxybutyrate in the metformin-containing arm of the METTEN study (*manuscript in preparation*), might have a significant impact on the design of future trials evaluating the potential of combining metformin with targeted therapy.

In summary, we have genotyped a subset of patients included in a neoadjuvant breast cancer trial to explore the effect of r*s11212617* variants on the clinical endpoint pCR, a powerful predictor of long-term outcome of patients with HER2-positive disease treated with neoadjuvant therapy with or without HER2-targeted agents (31–33). The present findings, although limited by the small effect size, suggest that further analyses using a larger number of breast cancer patients treated with metformin should verify whether a pharmacogenomic profile including the analysis of *ATM* SNP *rs11212617* genotype might deserve consideration as a predictive clinical biomarker to inform the personalized use of metformin in a cancer setting.

## Conclusions

Association with a significantly augmented pCR rate was found in metformin-treated breast cancer patients that have a “favorable” C allele-containing *ATM* SNP rs11212617 genotype. Because achievement of pCR is an appropriate surrogate for significantly improved long-term clinical outcomes in high-risk breast cancer subtypes (34), future studies validating this association of favorable *ATM* rs11212617 genotype with improvements in relapse-free survival after surgery in the METTEN study (and retrospective outcome analyses for other clinical trials) should definitely determine whether the *rs11212617 C* allele may lead to actionable modifications for prospective clinical planning in metformin-based anti-breast cancer approaches.

## Data Availability

The datasets generated and analyzed during the current study are available from the corresponding authors on reasonable request.

## Ethics Statement

The hospital (Dr. Josep Trueta Hospital, Girona, Spain) ethics committee (Clinical Investigation Ethic Committee, CIEC) and independent institutional review boards at each site participating in the METTEN study approved the protocol and any amendments. All procedures were in accordance with the ethical standards of the institutional research committees and with the 1964 Helsinki declaration and its later amendments or comparable ethical standards. Informed consent was obtained from all individual participants included in the study. The authors declared that they have no competing interests.

## Author Contributions

BM-C and JM: conceptualization, supervision, and funding acquisition; BM-C, MB, and JM: methodology; MB, JM, and EC: formal analysis and visualization; EC, SV, MF, SP, JD, IA, SM, JP-G, NB-L, CR-S, KA, SD, ML, AS, IM, GV, JC, and JJ: investigation; JB, EL-B, MG, SS, and XQ: resources; EC, SS, and MB: data curation; JM: writing-original draft preparation; JM, JP-G, EC, and BM-C: writing-review and editing; BM-C: project administration.

### Conflict of Interest Statement

The authors declare that the research was conducted in the absence of any commercial or financial relationships that could be construed as a potential conflict of interest.
