# Supplementary material for: The C Allele of ATM rs11212617 Associates With Higher Pathological Complete Remission Rate in Breast Cancer Patients Treated With Neoadjuvant Metformin
Source: Front Oncol. 2019 Mar 28;9:193. doi: 10.3389/fonc.2019.00193 (PMC6447648; doi:10.3389/fonc.2019.00193)
Supplement: Supplementary file 1 [file Data_Sheet_1.docx]

**The *C* allele of *ATM* rs11212617 associates with**

**higher pathological complete remission rate in**

**breast cancer patients treated with neoadjuvant metformin**

Elisabet Cuyàs, Maria Buxó, Maria José Ferri Iglesias, Sara Verdura, Sonia Pernas,

Joan Dorca, Isabel Álvarez, Susana Martínez, Jose Manuel Pérez-Garcia,

Norberto Batista-López, César A. Rodríguez-Sánchez, Kepa Amillano,

Severina Domínguez, Maria Luque, Idoia Morilla, Agostina Stradella, Gemma Viñas,

Javier Cortés, Jorge Joven, Joan Brunet, Eugeni López-Bonet, Margarita Garcia,

Samiha Saidani, Xavier Queralt Moles, Begoña Martin-Castillo, Javier A. Menendez

**SUPPLEMENTARY INFORMATION**

**Table S1. Distribution of ATM *rs11212617* genotype in treatment arms**

|  |  |  |
| --- | --- | --- |
|  |  | ***n (%)^a^*** |
| **Arm A** | *A/A* | 17 (51.5%) |
|  | *A/C,C/C* | 16 (48.5%) |
|  |  |  |
| **Arm B** | *A/A* | 20 (54.1%) |
|  | *A/C,C/C* | 17 (45.9%) |

^a^in each treatment arm

**Table S2. Association of ATM *rs11212617* genotype and pCR**

|  |  |  | | Unadjusted |  | Adjusted |  |
| --- | --- | --- | --- | --- | --- | --- | --- |
|  |  | **non-pCR *n (%)*** | **pCR *n (%)*** | **Odds ratio (95% CI)** | ***p*-value** | **Odds ratio (95% CI)** | ***p*-value** |
| **ATM *rs11212617*** | *A/A* | 18 (48.7%) | 19 (51.3%) | 1 |  | 1 |  |
|  | *A/C,C/C* | 10 (30.3%) | 23 (69.7%) | 2.18 (0.82–5.82) | 0.121 | 2.20 (0.82–5.88) | 0.118 |
|  |  |  |  |  |  |  |  |
| **Arm** | B | 14 (34.1.%) | 27 (65.9%) | 1 |  | 1 |  |
|  | A | 17 (44.7%) | 21 (55.3%) | 0.64 (0.26–1.59) | 0.337 | 0.80 (0.30–5.13) | 0.661 |

**Table S3. Adjusted association of the interaction between**

**ATM *rs11212617* genotype and pCR by treatment arm**

|  |  |  |  |
| --- | --- | --- | --- |
|  |  | **Odds ratio (95% CI)** | ***p*-value*** |
| **ATM *rs11212617*** | *A/A* | 1 |  |
|  | *A/C, C/C* | 0.59 (0.13–2.77) | 0.505 |
| **Arm** | B | 1 |  |
|  | A | 0.22 (0.05–0.98) | 0.046 |
| **Genotype × Arm** |  | 20.53 (1.97–213.79) | 0.011 |

*Adjusted by tumor size and hormone receptor status

**Table S4. Adjusted association between ATM *rs11212617* genotype and pCR in arm A**

|  |  |  |  |
| --- | --- | --- | --- |
|  |  |  |  |
| **Arm A** |  | **Odds ratio (95% CI)** | ***p*-value*** |
| **ATM *rs11212617*** | *A/A* | 1 |  |
|  | *A/C, C/C* | 28.88 (2.20–378.73) | 0.010 |

*Adjusted by tumor size and hormone receptor status

**Table S5. Adjusted association between ATM *rs11212617* genotype and pCR in arm B**

|  |  |  |  |
| --- | --- | --- | --- |
|  |  |  |  |
| **Arm B** |  | **Odds ratio (95% CI)** | ***p*-value*** |
| **ATM *rs11212617*** | *A/A* | 1 |  |
|  | *A/C, C/C* | 0.53 (0.11–2.50) | 0.425 |

*Adjusted by tumor size and hormone receptor status
